# The Anti-Inflammatory and Anti-Pruritus Mechanisms of Huanglian Jiedu Decoction in the Treatment of Atopic Dermatitis

**DOI:** 10.3389/fphar.2021.735295

**Published:** 2021-12-02

**Authors:** Yubin Xu, Saizhen Chen, Lingling Zhang, Guirong Chen, Jinguang Chen

**Affiliations:** ^1^ Department of Pharmacy, Taizhou Central Hospital (Taizhou University Hospital), Taizhou, China; ^2^ College of Pharmacy, Liaoning University of Traditional Chinese Medicine, Shenyang, China; ^3^ 67th Hospital of the Joint Logistics Support Force of the Chinese People’s Liberation Army, Dalian, China; ^4^ Department of Dermatology, Taizhou Central Hospital (Taizhou University Hospital), Taizhou, China

**Keywords:** atopic dermatitis, Huanglian Jiedu decoction, inflammation, pruritus, skin disease

## Abstract

Atopic dermatitis (AD) is a common chronic skin disease driven by a T-cell-mediated immune response, with inflammation and pruritus being its main clinical manifestations. Huanglian Jiedu decoction (HLJDT), which is an ancient Chinese medicine herbal formula derived from *Wai-Tai-Mi-Yao*, is a potentially effective treatment for AD. We aimed to clarify the anti-inflammatory and anti-pruritus mechanisms of HLJDT in AD treatment. We performed immunohistochemistry, Western blotting, reverse transcriptase-polymerase chain reaction, Luminex-based direct multiplex immunoassay, enzyme-linked immunosorbent assays, and flow cytometry to address the abovementioned aims. HLJDT significantly reduced clinical symptoms and ear swelling in AD-like mice by inhibiting the production of cytokines [histamine, interleukin (IL)-3, IL-4, IL-5, IL-13, IL-17A, IL-31, and IL-33], substance P (SP), transient receptor potential cation channel subfamily V member 1 (TRPV-1), and gastrin-releasing peptide (GRP). Additionally, HLJDT significantly suppressed the protein expression levels and positive cell percentage of CD28, CD80, CD86, CD207, CD326, MHCII, and OX40 in the lymphoid nodes. Moreover, HLJDT significantly suppressed mRNA and protein expression of tyrosine–protein kinase (JAK1), histamine H4 receptor, and IL-4Rα, as well as the protein expression of GRP, SP, and TRPV-1 in the root ganglion. Our findings indicate that HLJDT can treat AD by regulating the antigen presentation function of dendritic cells, weakening T-lymphocyte activation, and subsequently exerting anti-inflammatory and anti-pruritus effects.

## Introduction

Atopic dermatitis (AD) is a common, highly heritable chronic skin disease that is usually mediated by IgE and is clinically characterized by erythema, itch, eczema skin lesions, skin thickening, and inflammation (Badloe et al., 2020; Kuo et al., 2020). Worldwide, 15%–30% of children and 2%–10% of adults are affected by AD (Eichenfield et al., 2012). The exact pathogenesis of AD remains unclear; however, it is widely considered to be induced by the complex interaction of skin barrier dysfunction and skin inflammation, with the involvement of environmental, genetic, and immunological factors; moreover, immunological abnormality is considered a key link (Bonamonte et al., 2019; Eichenfield et al., 2012; Fenner and Silverberg, 2018). The main clinical manifestations of AD are inflammation and pruritus.

AD involves an aberrant immune response involving several crosslinks, including antigen recognition, processing, and presentation in Langerhans cells (LCs) and dendritic cells (DCs); Th2-dominant abnormal immune responses; compromised function of regulatory T cells; IgE overexpression; and increased eosinophil levels (Guttman-Yassky et al., 2017; Peng and Novak, 2015). Abnormal cytokine secretion induced by Th1/Th2 imbalance is crucially involved in AD development. Specifically, Th2 cells are principally involved during the acute and chronic phases of AD; contrastingly, Th1 cell-mediated inflammatory reactions are crucially involved in the chronic AD phase (Gittler et al., 2012). The main effector cells of chronic skin lesions in AD are eosinophils and mast cells, which mediate numerous inflammatory molecules, including histamine, leukotriene, and interleukin, causing pruritus and mossy skin lesions in patients with AD. However, other inflammatory products, including kinin, neuropeptide, and platelet-activating factor may be involved in AD progression (Cheung et al., 2010; de la and Sidbury, 2020; Raap et al., 2006). Additionally, interleukin (IL)-31 is closely associated with AD-induced pruritus, which synergistically works with histamine H4 receptor (HRH4) to aggravate pruritus and skin lesions (Otsuka et al., 2011). A recent study demonstrated that IL-4Rα and tyrosine-protein kinase (JAK1) are key indicators of inflammatory and non-inflammatory chronic pruritus, respectively; moreover, it showed that IL-4 can significantly worsen pruritus symptoms caused by indicators such as histamine (Oetjen et al., 2017). Accordingly, anti-inflammatory and anti-pruritus effects are key factors in AD treatment.

Huanglian Jiedu decoction (HLJDT) is an ancient Chinese medicine herbal formula derived from *Wai-Tai-Mi-Yao*. HLJDT is known to possess therapeutic effects of clearing heat and detoxification in Chinese medicine practice and is widely used in skin diseases, such as eczema and AD (Chen GR. et al., 2016; Ko and Baek, 2012; Yang et al., 2017). HLJDT is composed of four Chinese medicines, including *Coptis chinensis* Franch (Huanglian, whose rootstocks have medicinal use), *Scutellaria baicalensis* Georgi (Huangqin, whose root has medicinal use), *Phellodendron amurense* Rupr. (Huangbai, whose bark has medicinal use), and *Gardenia jasminoide* J. Ellis (Zhizi, whose fruit has medicinal use), at a ratio of 3:2:2:3. It exerts therapeutic effects of heat clearing and detoxification against the fire-toxicity syndrome (Chen et al., 2017). Modern pharmacological studies have confirmed that HLJDT possesses antipyretic, anti-inflammatory, anti-bacterial, anti-ulcer, and hemostasis properties, which can be clinically applied in the treatment of AD, sepsis, multi-organ dysfunction syndrome, hypertension, cerebrovascular disease, pneumonia, and meningitis (Fang, 2015; Xia et al., 2018). Although HLJDT can allow multi-target and multi-pathway treatment of AD (Chen Y. et al., 2016; Chen et al., 2020; Kobayashi et al., 2004), the anti-inflammatory and anti-pruritus mechanisms of HLJDT for AD treatment remain unclear.

This study aimed to determine whether AD treatment by HLJDT mainly involved anti-inflammatory and anti-pruritus activities, as well as to systematically explore these anti-inflammatory and anti-pruritic mechanisms.

## Materials and Methods

### Materials

*Coptis chinensis* Franch, *Scutellaria baicalensis* Georgi, *Phellodendron amurense* Rupr, and *Gardenia jasminoides* J. Ellis were purchased from the Hangzhou Mintai (Bozhou) Chinese Herbal Medicine Co. Ltd. (Zhejiang, China) and identified by professor Rui-Ping Huang [Department of Pharmacy, Taizhou Central Hospital (Taizhou University Hospital)] following the Chinese Pharmacopoeia (2015 Edition). Voucher specimens of the aforementioned herbs were deposited in the Department of Pharmacy of Taizhou Central Hospital (Taizhou University Hospital), Taizhou, Zhejiang, with reference no. Xu1-4. We purchased 2,4-dinitrofluorobenzene (DNFB) from the Shanghai McLean Biochemical Technology Co., Ltd. (Shanghai, China). All the other chemicals used were of analytical grade.

### Huanglian Jiedu Decoction Preparation

HLJDT decoction was prepared by the Pharmacy Department of the General Hospital of Liaoning University of Traditional Chinese Medicine (300 ml per bottle, batch no. 2020054). In brief, 600 g of the mixed Chinese medicine (*Coptis chinensis* Franch, *Scutellaria baicalensis* Georgi, *Phellodendron amurense* Rupr., and *Gardenia jasminoides* J. Ellis, with a ratio of 3:2:2:3) was measured in proportion. Water was used as the solvent to extract twice for 1 h each; the filtrate was collected with a treatment of hot filtration and evaporated to obtain the appropriate concentrated soup. Subsequently, the quality of HLJDT was evaluated through high liquid chromatography, and the contents of berberine and baicalin in HLJDT were determined to be 4.51 and 7.92 mg/g, respectively; the HPLC fingerprinting was constructed as quality control for HLJDT as previously described (Chen Y. et al., 2016). Furthermore, we determined the chemical composition of HLJDT through UPLC-Q-TOF-MS/MS, which confirmed that alkaloids, flavonoids, organic acids, glycosides, and iridoids were the main components, with some being validated by matching their retention time and accurate mass measurement with those in the available reference standards (Table 1). Supplementary Table S1 presents the raw total data.

**TABLE 1:** Ingredients from Huanglian Jiedu decoction (HLJDT) validated by available reference standard.

No	R.T. (min)	*m/z*	Ion mode	Elemental composition	Identification results	Classification
1	8.35	269.0455	Negative	C15H10O5	Baicalein	Flavonoids
2	9.81	253.0506	Negative	C15H10O4	Chrysin	Flavonoids
3	10.28	343.0818	Negative	C18H16O7	Eupatilin	Flavonoids
4	9.72	283.0612	Negative	C16H12O5	Wogonin	Flavonoids
5	7.80	459.0923	Negative	C22H20O11	Wogonoside	Flavonoids
6	7.35	447.0916	Positive	C21H18O11	Baicalin	Flavonoids
7	10.02	315.0859	Positive	C17H14O6	3,7-Dihydroxy-3′,4′-dimethoxyflavone	Flavonoids
8	10.10	375.1072	Positive	C19H18O8	Casticin	Flavonoids
9	3.77	341.1622	Negative	C20H24NO4	Phellodendrine	Alkaloids
10	2.45	270.1132	Negative	C16H17NO3	Higenamine	Alkaloids
11	5.90	322.1069	Positive	C19H15NO4	Berberrubine	Alkaloids
12	6.08	352.1176	Positive	C20H19NO6	Egenine	Alkaloids
13	6.57	338.1379	Positive	C20H19NO4	Dihydroberberine	Alkaloids
14	7.09	336.1221	Positive	C20H18NO4	Epiberberine	Alkaloids
15	7.28	336.1227	Positive	C20H18NO4	Berberine	Alkaloids
16	6.45	320.0913	Positive	C19H14NO4	Coptisine	Alkaloids
17	6.95	352.1528	Positive	C21H22NO4	Palmatine	Alkaloids
18	8.40	260.0914	Positive	C14H13NO4	Skimmianine	Alkaloids
19	0.58	191.0558	Negative	C7H12O6	Quinic acid	Organic acids
20	3.12	353.0873	Negative	C16H18O9	4-O-Caffeoylquinic acid	Organic acids
21	2.90	353.0874	Negative	C16H18O9	Chlorogenic acid	Organic acids
22	0.81	191.0201	Negative	C6H8O7	Citric acid	Organic acids
23	0.63	134.0214	Negative	C4H6O5	DL-Malic acid	Organic acids
24	2.67	153.0194	Negative	C7H6O4	Gentisic acid	Organic acids
25	1.64	198.0530	Negative	C9H10O5	Salvianic acid A	Organic acids
26	1.78	373.1135	Negative	C16H22O10	Geniposidic acid	Organic acids, iridoids
27	3.82	225.0771	Negative	C11H14O5	Genipin	Iridoids
28	2.28	404.1314	Negative	C17H24O11	Gardenoside	Glycosides, iridoids
29	3.82	433.1340	Negative	C17H24O10	Geniposide	Glycosides, iridoids
30	7.36	593.1856	Positive	C28H32O14	Linarin	Glycosides

### Animal Experiments and Sample Collection

We purchased specific pathogen-free (SPF) male C57BL/6 mice (age: 4–6 weeks; weight: 20 ± 2 g) from the Shrek Jinda Laboratory Animals Co., Ltd. [Hunan, China; laboratory animal license, SCXK (Su): 2016–0010]. The animals were kept under SPF laboratory conditions; moreover, all animal experimental procedures were approved by the ethics committee of the institution (ID number: SHDSYY-2020-9001).

Based on our preliminary findings, C57BL/6 mice were randomly divided into five groups (each *n* = 8) as follows: the control group, model group, and three HLJDT groups (3.2, 6.4, and 12.8 g/kg, with the dosage being calculated using crude drugs equivalent to one, two, and four times the human dose, respectively). DNFB was dissolved in acetone/olive oil (4:1) to prepare the proper solutions before administration to the mice. The procedure was performed as previously described, with slight modifications (Chen et al., 2020). Briefly, an electric shaver was used to remove hair from the abdominal and back skin (area of about 2 × 2 cm^2^) of the mice on the first day. Next, 200 μl of 1% DNFB was used to sensitize the abdominal skin on days 1, 4, and 7 (once a day). Subsequently, the model and HLJDT groups were administered with 200 μl of 0.5% DNFB on the back skin and right ear on days 14, 17, 19, 22, 24, 27, and 29 (once a day), with the control group being smeared with an equal volume of DNFB matrix solution at the same time points. Next, the HLJDT groups were intragastrically administrated with 3.2, 6.4, and 12.8 g/kg HLJDT once a day for 16 days, while the control and model groups received an equal saline volume at the same time points. After the last administration, the mice were anesthetized using pentobarbital sodium followed by blood sample collection to obtain serum. Finally, the animals were sacrificed, followed by the collection of the dorsal root ganglion and lymphoid nodes. The obtained tissues and serum were placed in a refrigerator at −80°C for subsequent analyses. Some of the tissues were cut and immersed in 4% paraformaldehyde for immunohistochemical analysis.

### Evaluation of Dorsal Skin Lesions and Ear Swelling

We evaluated dorsal skin lesions according to the following clinical criteria (Choi et al., 2018): erythema, edema/papules, epidermal stripping/scratches, and scales (dry), which were graded and scored as follows: no symptoms (0), mild (1), medium (2), and severe (3). Vernier calipers were used to measure the thickness of the left and right ears of each mouse, with the average value of triplicate measurements being calculated to determine between-ear differences in thickness for ear swelling evaluation.

Cytokines (histamine, IgE, IL-3, IL-4, IL-5, IL-13, IL-17A, IL-31, and IL-33), gastrin-releasing peptide (GRP), substance P (SP), and transient receptor potential cation channel subfamily V member 1 (TRPV-1) were determined in the serum of mice.

Mouse Custom ProcartaPlex 8-plex kit (Invitrogen, USA) was used to detect IL-3, IL-4, IL-5, IL-13, IL-17A, IL-31, and IL-33 levels using the Luminex-based direct immunoassay platform (Luminex 200, Thermo Fisher Scientific), following the instructions of the manufacturer. Moreover, the data were analyzed using ProcartaPlex Analyst 1.0 software. Enzyme-linked immunosorbent assays (ELISA) kits were purchased from Cloud-Clone Corp (Wuhan, China) for measuring the histamine, IgE, GRP, SP, and TRPV-1 levels.

### Reverse Transcriptase-Polymerase Chain Reaction Analysis

Reverse transcriptase-polymerase chain reaction (RT-PCR) analysis was used to detect HRH4, IL-4Rα, JAK1, GRP, SP, TRPV-1, and β-actin mRNA expression. Total RNA was isolated from the dorsal root ganglion tissues using TRIzol^®^ reagent (Invitrogen; Thermo Fisher Scientific, Inc.). Subsequently, we conducted reverse transcription and produced cDNAs using the HiScript II Q RT SuperMix for qPCR (+gDNA wiper) (R223-01, Vazyme, Inc, Nanjing, China), which were subjected to RT-PCR analysis using 2× SYBR Green PCR Master Mix (A4004M, Lifeint, Xiamen, China) on CFX Connect™ PCR instrument (Bio-Rad Laboratories, Shanghai, China) according to the protocol of the manufacturer. The primers were as follows: HRH4 specific primer (forward: TGA​CTT​CCT​CGT​GGG​TTT​G, reverse: ATT​GTA​GAC​AGA​TGC​GGT​GC), IL-4Rα specific primer (forward: GGA​GGA​GGA​AGA​AGA​TGA​GAT​AG, reverse: CCA​ACA​AGT​CGG​AAA​ACA​GG), JAK1 specific primer (forward: GGC​GTT​CTG​TGC​TAA​AAT​GA, reverse: AGG​GCG​AAG​AGG​TTG​TGA​C), GRP specific primer (forward: TGG​GCT​GTG​GGA​CAC​TTA​AT, reverse: GCT​TCT​AGG​AGG​TCC​AGC​AAA), SP specific primer (forward: CAA​AGA​GCG​CCC​AGC​AAG​TGC, reverse: TGC​TCA​AAG​GGC​TCC​GGC​ATT), TRPV-1 specific primer (forward: TCA​CCG​TCA​GCT​CTG​TTG​TC, reverse: GAT​CAT​AGA​GCC​TTG​GGG​GC), and β-actin specific primer (forward: AGG​GAA​ATC​GTG​CGT​GAC, reverse: CAT​ACC​CAA​GAA​GGA​AGG​CT). After normalization to the β-actin level, we calculated the expression levels of target mRNAs.

### Western Blot Assay

Western blot analysis was performed as previously described, with slight modifications (Chen et al., 2021). Briefly, the dorsal root ganglion tissues were homogenized in 200 μl of RIPA lysis buffer and extracted for 30 min on ice; subsequently, they were centrifuged at 12,000 rpm for 10 min at 4°C. We collected and quantified the supernatant using a BCA protein assay kit (Beijing ComWin Biotech Co., Ltd. China).

Next, equal protein amounts of the different samples were separated and electroblotted through 10% sodium dodecyl sulfate-polyacrylamide gel electrophoresis and polyvinylidene difluoride (PVDF) membranes. After blocking using 3% nonfat milk overnight, the PVDF membranes were incubated with the primary antibody at 4°C for 3 h, followed by rinsing with TBST thrice for 10 min each. Next, the PVDF membranes were incubated with a secondary antibody solution for 2 h at room temperature. Subsequently, they were washed and visualized using an ECL (RJ239676, Thermo) method through a high-sensitivity chemiluminescence imaging system [Chemi DocTM XRS, Berle Life Medicine Products (Shanghai) Co., Ltd, China]. Antibodies for Western blot analysis included anti-H4R (Omnimabs # OM209489), anti-JAK1 (Proteintech # 66,466-1-ap), anti-p-JAK1 (Affinity # AF 2012), anti-IL-4Rα (Affinity # DF8567), and anti-β-actin (Abcam # ab8226) antibodies.

### Immunohistochemical Staining

Immunohistochemical staining was performed as previously described, with slight modifications (You et al., 2020). Briefly, tissues fixed in 4% polyformaldehyde solution were sliced using a paraffin-sectioning machine, stained using streptavidin–biotin complex immunohistochemistry kit, dehydrated with a gradient ethanol solution, hyalinized with xylene, sealed with neutral gum, and finally analyzed using a fluorescence microscope Olympus BX51 (Japan).

### Analysis of Flow Cytometry

Lymph node tissues were ground and disrupted using a glass homogenizer in an ice bath, with a single-cell suspension being prepared using a 200-mesh filter. The cell concentration was adjusted to 1 × 10^7^ times/ml; subsequently, we added 200 μl of cell suspension with 5 μl of PE-CD28/CD80/CD207 fluid antibody, followed by incubation for 30 min in a water bath at a constant temperature of 37°C. After incubation, the cells were resuspended in 200 μl of phosphate-buffered saline and incubated with 5 μl of FITC-OX40/CD86/MHC-II/CD326 fluid antibody at the same condition. All the antibodies were purchased from BD Pharmingen. Phycoerythrin and fluorescein isothiocyanate signals were analyzed using CytoFlex (Beckman, USA). DC populations were selected according to the forward- and side-scatter.

### Statistical Analysis

Data were presented as mean ± standard deviation. Statistical analyses were performed by one-way analysis of variance using the statistical software SPSS 20.0 (Chicago, IL, USA). Statistical significance was set as *p* < 0.05.

## Results

### Evaluation of Dorsal Skin Lesions and Ear Swelling of 2,4-Dinitrofluorobenzene-Treated Mice

**FIGURE 1:**
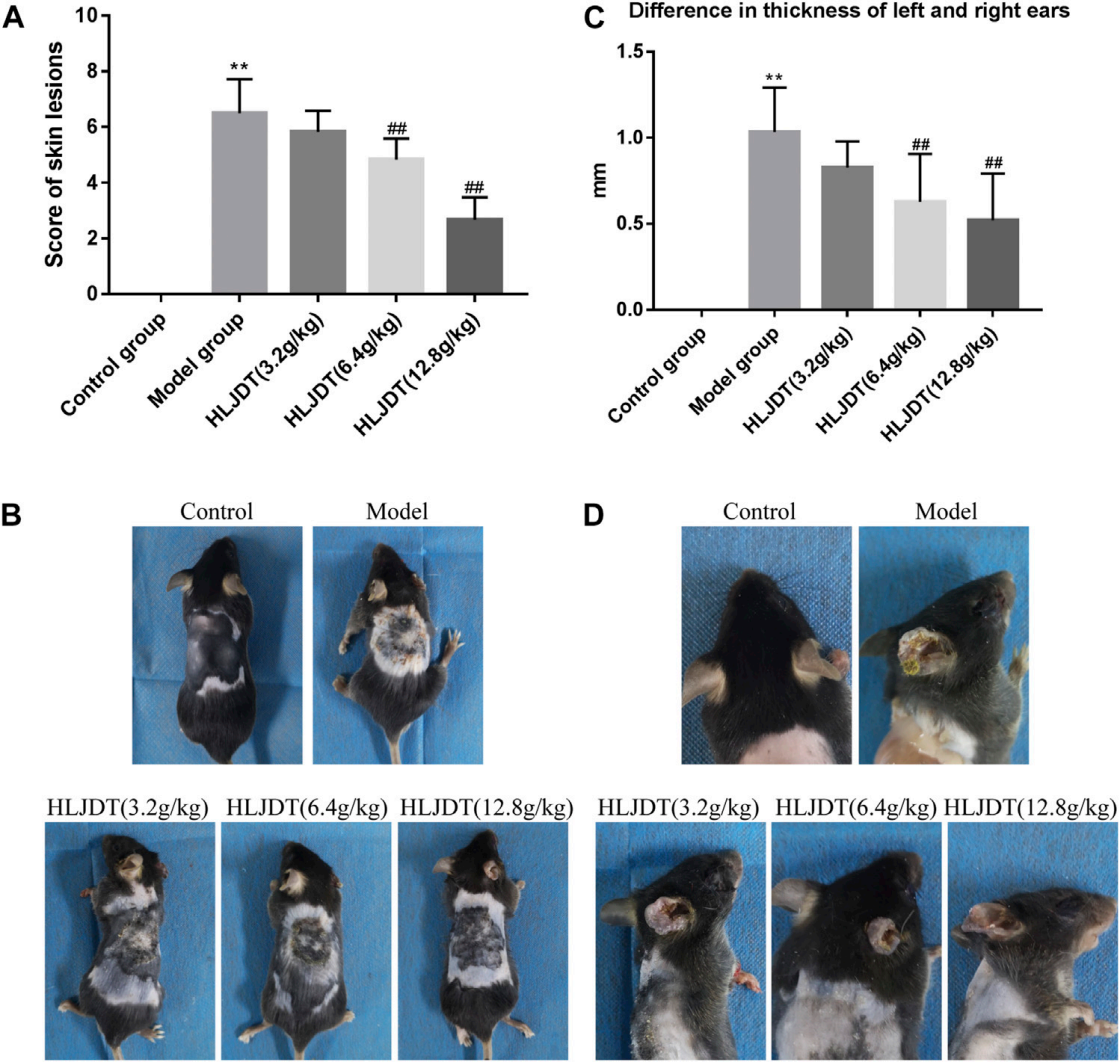
The effects of Huanglian Jiedu decoction (HLJDT) on dorsal skin lesions and ear swelling in 2,4-dinitrofluorobenzene (DNFB)-treated mice. **(A)** The score of dorsal skin lesions in each group. **(B)** Clinical features of dorsal skin lesions in each group. **(C)** The difference in thickness of left and right ears of mice in each group. **(D)** Ear swelling of mice in each group. *n* = 8 for all groups. Clinical features of dorsal skin lesions in mice included erythema, edema/papules, epidermal stripping/scratches, and scales (dry). ^**^ *p* < 0.01 vs. control group; ^##^ *p* < 0.01 vs. model group.

AD-like skin symptoms and between-ear differences in thickness in each mouse were scored after completing drug treatment. The severity of each symptom and ear swelling was evaluated through comparison with the reference photographs. As shown in Figures 1A, B, compared with the control group, the model group showed edema, epidermal stripping, and scales, with a significant increase in the clinical score (*p* < 0.01), which was significantly reduced by 6.4 and 12.8 g/kg of HLJDT (*p* < 0.01). Compared with the control group, the model group showed significantly higher between-ear thickness differences (*p* < 0.01); furthermore, 6.4 g/kg and 12.8 g/kg HLJDT significantly inhibited the DNFB-induced ear swelling (*p* < 0.01) (Figures 1C, D).

### Huanglian Jiedu Decoction Effects on Serum Histamine, IgE, Interleukin-3, Interleukin-4, Interleukin-5, Interleukin-13, Interleukin-17A, Interleukin-31, Interleukin-33, Gastrin-Releasing Peptide, Substance P, and Transient Receptor Potential Cation Channel Subfamily V Member 1 Levels

**FIGURE 2:**
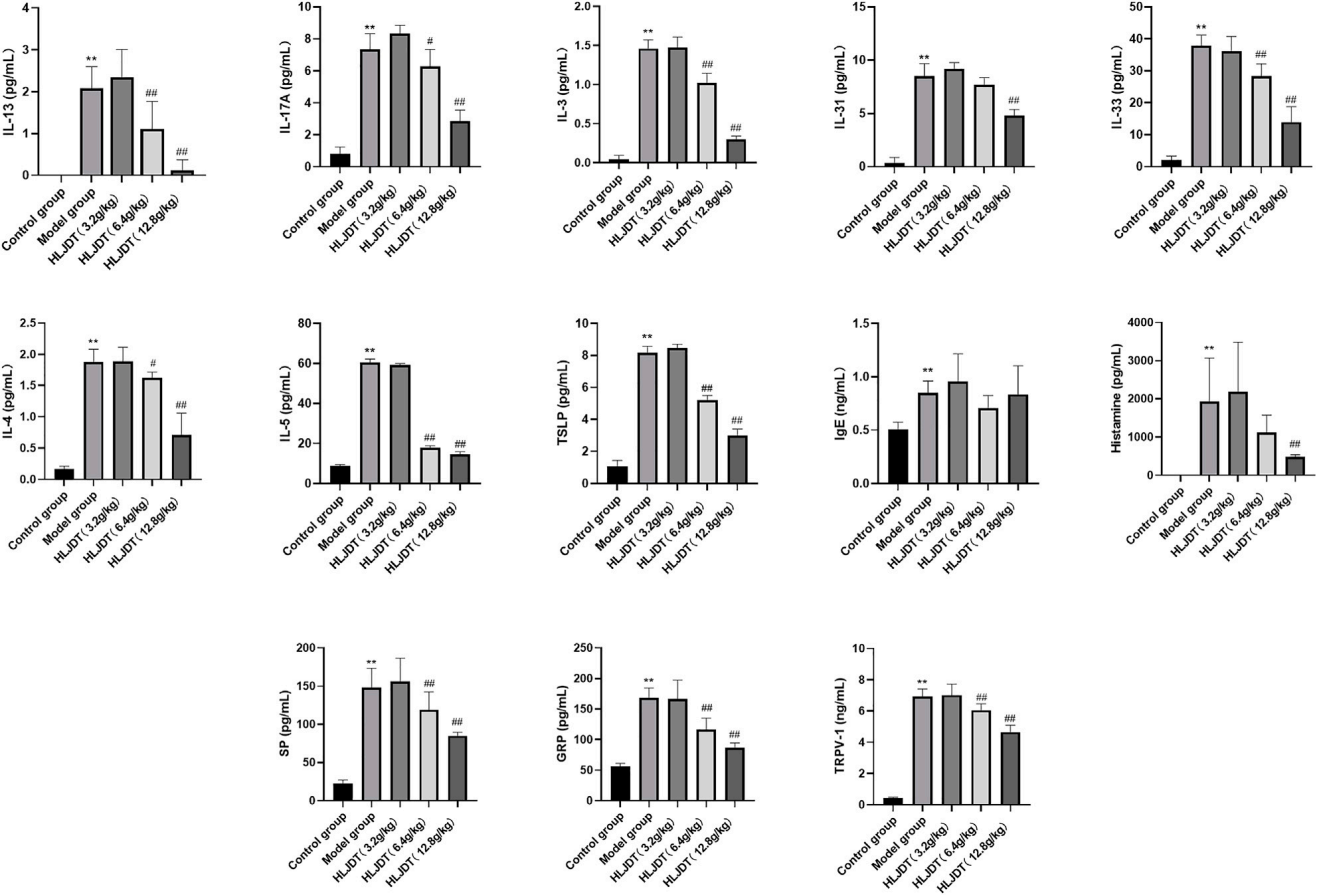
Effects of HLJDT on serum histamine, IgE, interleukin (IL)-3, IL-4, IL-5, IL-13, IL-17A, IL-31, IL-33, gastrin-releasing peptide (GRP), substance P (SP), and transient receptor potential cation channel subfamily V member 1 (TRPV-1) levels. ^**^ *p* < 0.01 vs. control group; ^#^ *p* < 0.05, ^##^ *p* < 0.01 vs. model group. The levels of IL-3, IL-4, IL-5, IL-13, IL-17A, IL-31 and IL-33 were detected through the Luminex-based direct immunoassay platform, the levels of histamine, IgE, GRP, SP and TRPV-1 were detected by enzyme-linked immunosorbent assays (ELISA) method.

Luminex-based direct multiplex immunoassay and ELISA revealed significantly increased serum histamine, IgE, IL-3, IL-4, IL-5, IL-13, IL-17A, IL-3,1 and IL-33 levels in the model group than in the control group (*p* < 0.01), which was indicative of abnormal cytokine secretion mainly related to inflammation and pruritus in the model group. Notably, 12.8 g/kg HLJDT inhibited the abnormal secretion of the aforementioned cytokines (*p* < 0.01) except for IgE (*p* > 0.05) (Figure 10A, see *Discussion* section); furthermore, 6.4 g/kg HLJDT only significantly decreased IL-4 and IL-17A levels (*p* < 0.05), IL-3, IL-5, IL-13, and IL-33 (*p* < 0.01) compared with the levels in the model group. Finally, 3.2 g/kg HLJDT had no effect in the model group (Figure 2).

### Huanglian Jiedu Decoction Effects on Tyrosine–Protein Kinase, Histamine H4 Receptor, Interleukin-4αR, Gastrin-Releasing Peptide, Substance P, and Transient Receptor Potential Cation Channel Subfamily V Member 1 mRNA Expressions in the Dorsal Root Ganglion

**FIGURE 3:**
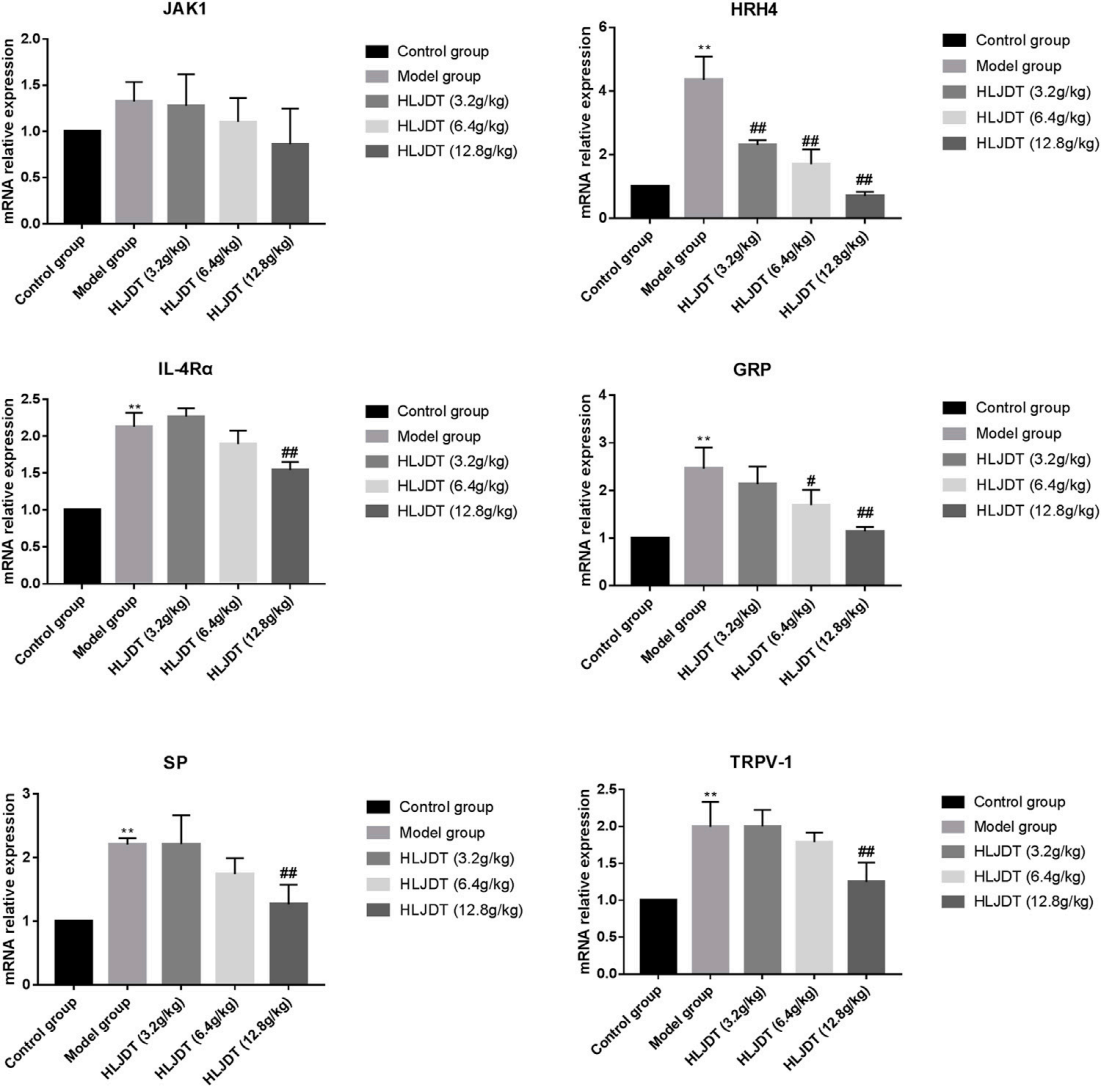
Effects of HLJDT on tyrosine–protein kinase (JAK1), histamine H4 receptor (HRH4), IL-4αR, GRP, SP, and TRPV-1 mRNA expressions in the dorsal root ganglion. ^**^ *p* < 0.01 vs. control group; ^#^ *p* < 0.05, ^##^ *p* < 0.01 vs. model group.

To further investigate the anti-pruritus mechanisms underlying HLJDT, we analyzed pruritus-related markers (i.e., JAK1, HRH4, IL-4αR, GRP, SP, and TRPV-1) from the dorsal root ganglion. The mRNA expression levels of all pruritus-related markers, except JAK1, were significantly upregulated in the model group, which were downregulated by 12.8 g/kg HLJDT. Additionally, 6.4 g/kg HLJDT downregulated HRH4 and GRP mRNA expressions, while 3.2 g/kg HLJDT only downregulated HRH4 mRNA expression. (Figure 3).

### Huanglian Jiedu Decoction Effects on Tyrosine–Protein Kinase, Histamine H4 Receptor, Interleukin-4αR, and p-JAK1 Protein Expressions in the Dorsal Root Ganglion

**FIGURE 4:**
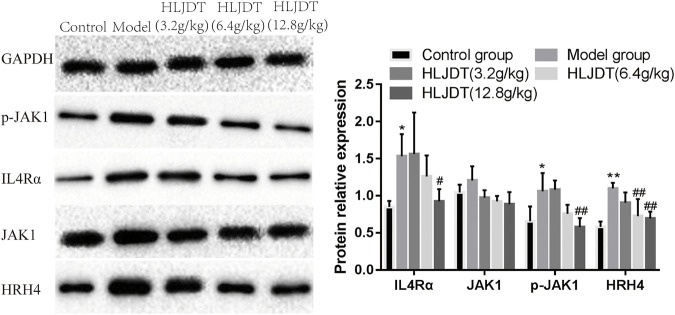
Effects of HLJDT on JAK1, p-JAK1, HRH4, and IL-4αR protein expressions in the dorsal root ganglion. ^*^ *p* < 0.05, ^**^ *p* < 0.01 vs. control group; ^#^ *p* < 0.05, ^##^ *p* < 0.01 vs. model group.

The model group showed significantly upregulated protein expressions of p-JAK1, HRH4, and IL-4αR, which were significantly downregulated by 12.8 g/kg HLJDT. Furthermore, 6.4 g/kg of HLJDT could downregulate HRH4 protein expression, while 3.2 g/kg HLJDT had no effect (Figure 4) (The original Western blot photos are shown in the Supplementary Materials).

### Immunohistochemistry Analysis of p-JAK1, Histamine H4 Receptor, Interleukin-4αR, Gastrin-Releasing Peptide, Substance P, and Transient Receptor Potential Cation Channel Subfamily V Member 1 Expression in the Dorsal Root Ganglion

**FIGURE 5:**
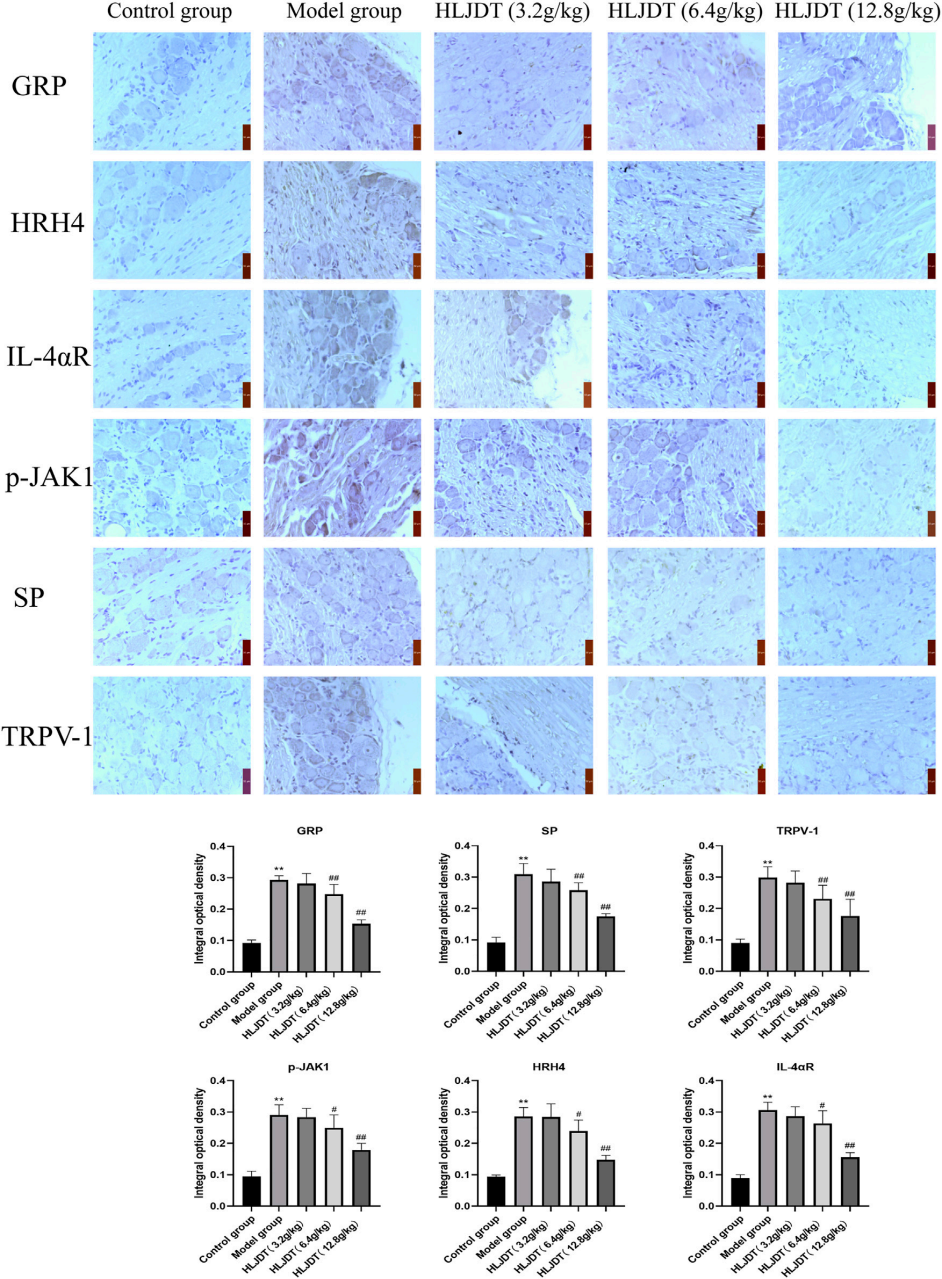
Effects of HLJDT on p-JAK1, HRH4, IL-4αR, GRP, SP, and TRPV-1 protein expressions in the dorsal root ganglion by immunohistochemistry. The positive expression of indices showed brown-yellow granules. The integral optical density of indices from each group were presented as the mean ± SEM, ^**^ *p* < 0.01 vs. control group; ^#^ *p* < 0.05, ^##^ *p* < 0.01 vs. model group.

As shown in Figure 5, compared with the control group, the model group showed a strongly increased protein expression of p-JAK1, HRH4, IL-4αR, GRP, SP, and TRPV-1 (*p* < 0.01). Compared with the model group, the 6.4- and 12.8-g/kg HLJDT groups showed weakly positive expression of p-JAK1, HRH4, IL-4αR, GRP, SP, and TRPV-1 proteins; moreover, there was a significant decrease in the number of positive cells (*p* < 0.05 or *p* < 0.01). Although this result was consistent with the RT-PCR and Western blot, 12.8 g/kg HLJDT had a more stable effect on AD than the 6.4 g/kg of HLJDT.

### Immunohistochemistry Analysis to Detect Expression of CD28, CD80, CD86, CD207, CD326, MHCII, and OX40 in the Lymphoid Nodes

**FIGURE 6:**
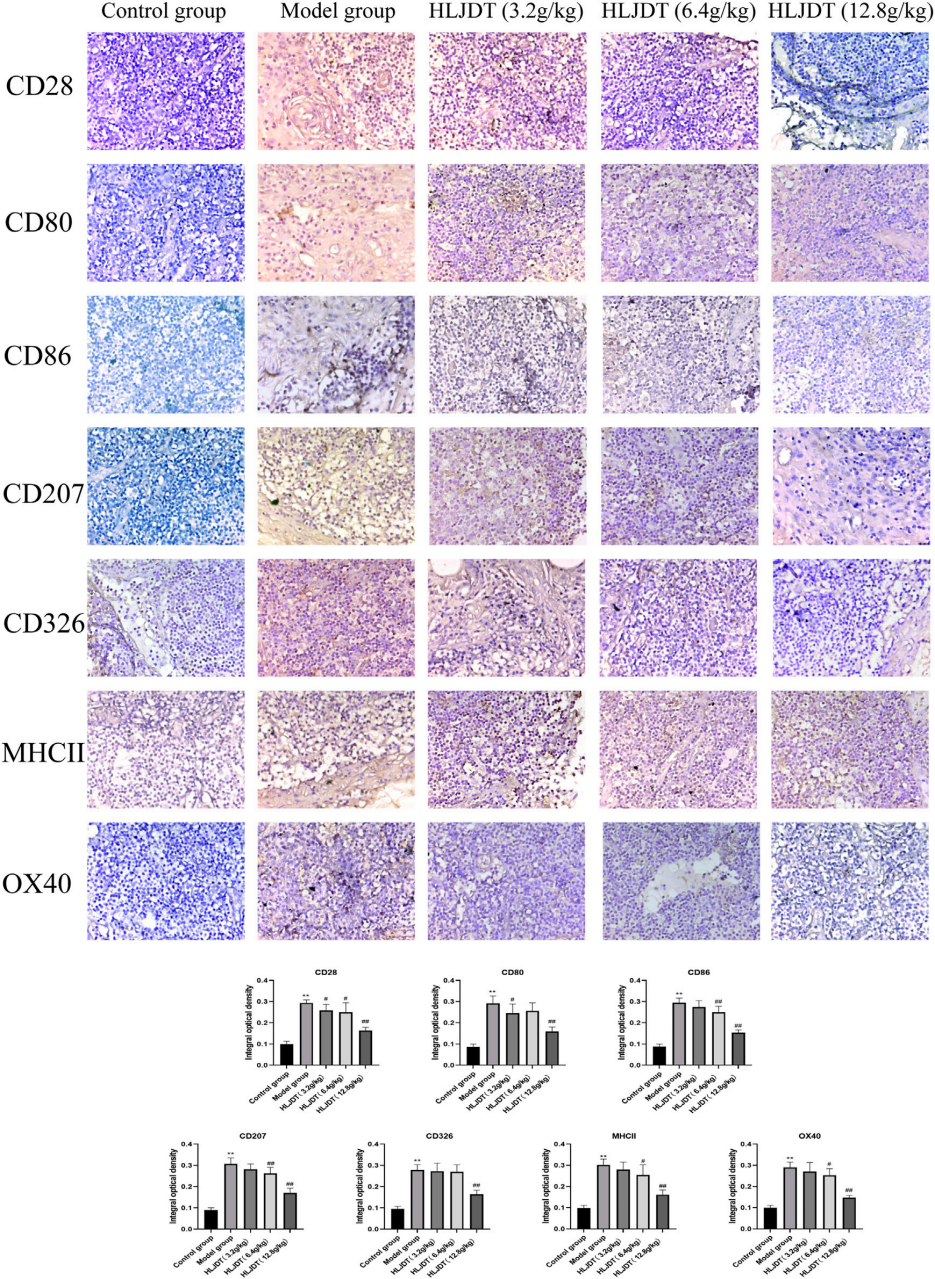
Effects of HLJDT on CD28, CD80, CD86, CD207, CD326, MHCII, and OX40 protein expressions in the lymphoid nodes by immunohistochemistry. The positive expression of indices showed brown-yellow granules. The integral optical density of indices from each group were presented as the mean ± SEM, ^**^ *p* < 0.01 vs. control group; ^#^ *p* < 0.05, ^##^ *p* < 0.01 vs. model group.

As shown in Figure 6, compared with the control group, the model group showed stronger positive expression of CD28, CD80, CD86, CD207, CD326, MHCII, and OX40 proteins (*p* < 0.01), which were significantly downregulated by 12.8 g/kg HLJDT (*p* < 0.01). Additionally, 3.2 g/kg of HLJDT downregulated CD28 and CD80 expressions, while 6.4 g/kg of HLJDT downregulated CD28, CD86, CD207, MHCII, and OX40 expressions compared with the expression levels in the model group (*p* < 0.05 or *p* < 0.01). Taken together, 12.8 g/kg HLJDT had the strongest anti-AD effect.

### Flow Cytometry Analysis of the Percentage of Denditric Cells Positive for CD28, CD80, CD86, CD207, CD326, MHCII, and OX40 in the Lymphoid Nodes

**FIGURE 7:**
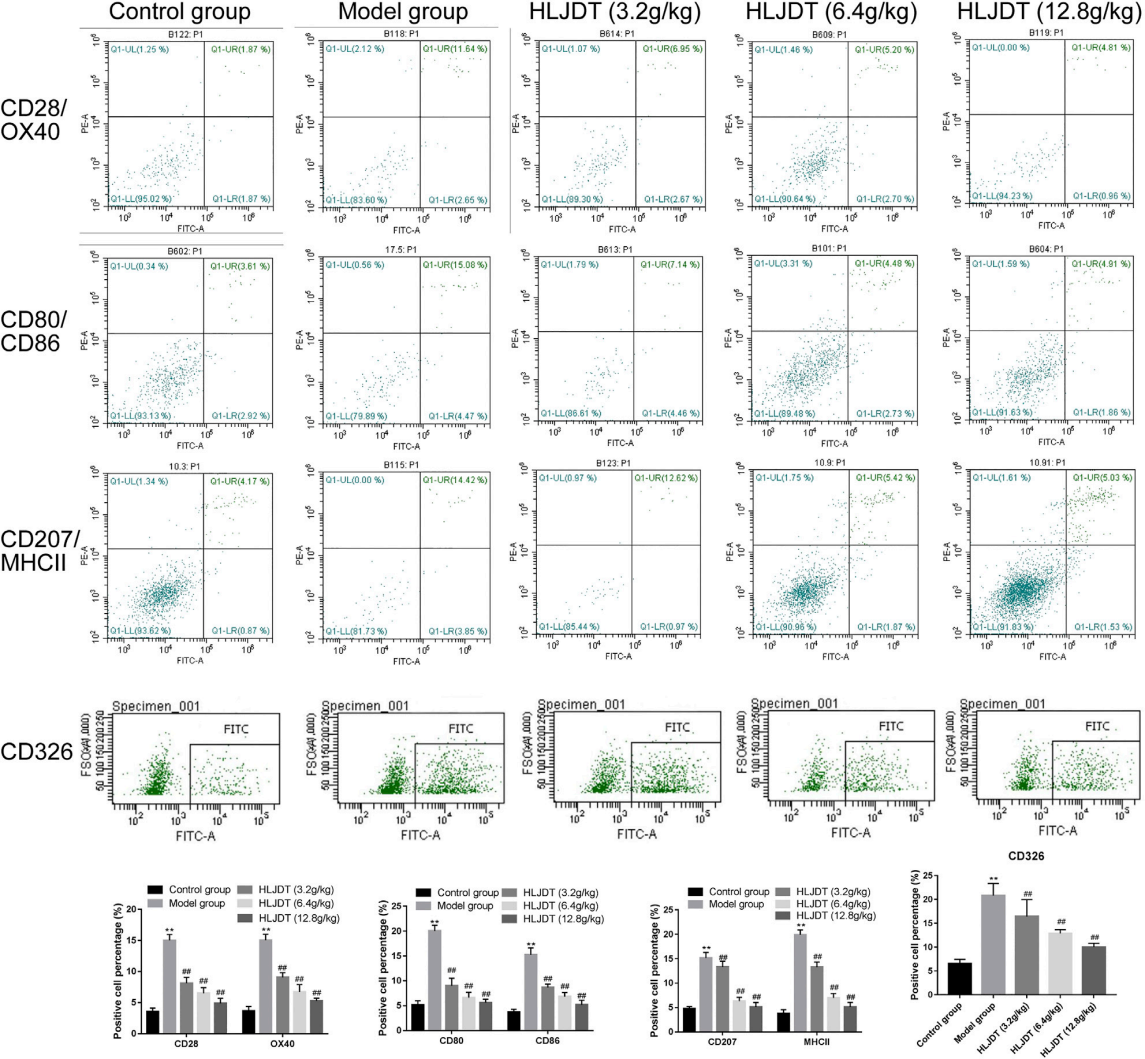
The percentage of positive cells for CD28, CD80, CD86, CD207, CD326, MHCII, and OX40 on dendritic cells (DCs) in the lymphoid nodes. The percentage of positive cells of indicators from each group were presented as the mean ± SEM, ^**^ *p* < 0.01 vs. control group; ^#^ *p* < 0.05, ^##^ *p* < 0.01 vs. model group.

As shown in Figure 7, the percentage of positive cells for CD28, CD80, CD86, CD207, CD326, MHCII, and OX40 on DCs in the lymphoid nodes were higher in the model group than in the control group (*p* < 0.01), which were significantly reduced by 3.2, 6.4, and 12.8 g/kg of HLJDT (*p* < 0.01). Furthermore, 12.8 g/kg HLJDT was the most efficient.

## Discussion

HLJDT has good therapeutic effects on AD (Chen et al., 2020), which is a recurrent chronic inflammatory skin disease mainly characterized by inflammation and pruritus. This study systematically analyzed the anti-AD mechanisms of HLJDT from these two perspectives.

In the skin, DCs are important innate immune cells that process and present antigens to T cells or other cells to initiate an immune response, which is crucially involved in immune system dysregulation (Klose and Artis, 2016; Scibior et al., 2019). There are two types of DCs: immature and mature. LCs are immature DCs and are the only DC subgroup present in the *epidermis*, with different functions in different skin regions (West and Bennett, 2017). Epidermal LCs are mainly involved in antigen phagocytosis, while those in the dermis and drainage lymph nodes mainly show antigen presentation function (Zhang et al., 2016). Moreover, they all express the C-type lectin CD207 (langerin) (Valladeau et al., 2000), which promotes skin allergic reactions and plays a role in the non-classical antigen processes (e.g., antigen recognition and phagocytosis), formation of Birbeck granules, transport to local lymphoid tissues, and induction of LC maturation (Wang et al., 2008). We found that the CD207 expression in the lymphoid nodes of AD mice was downregulated by 6.4 and 12.8 g/kg of HLJDT; moreover, LCs mediate epidermal sensitization by CD207, which stimulates the allergic response that causes AD development (Lee et al., 2012). This indicates that HLJDT can prevent AD development by inhibiting CD207 expression.

Epidermal LCs gradually differentiate and mature with upregulated expression of various costimulatory molecules (CD80, CD86, MHCⅡ, etc.) during their migration to draining lymph nodes after antigen uptake, which induces AD by stimulating T-lymphocyte activation (Netea et al., 2005). MHC-Ⅱ is a highly polymorphic transmembrane glycoprotein that can present LC-processed antigens to T-lymphocyte receptors for differentiation and activation (Romani et al., 2010). CD80 can promote T-cell differentiation to Th1, while CD86 can induce Th2-mediated humoral immune responses, which are crucially involved in the immune response of autoimmune diseases (Sharpe, 2009). Otherwise, CD80 and CD86 costimulatory molecules are widely expressed on antigen-presenting cells (Trzupek et al., 2020). OX40 (CD134), which is an early marker of T-cell activation, and its cognate ligand OX40L (mainly expressed in professional antigen-presenting cells, including B cells, DCs, and macrophages) are crucial costimulatory molecules in the TNFR/TNF superfamily (Cederbrant and Hultman, 2000; Rojas et al., 2020). Recent studies have described the involvement of OX40 and OX40L in CD4^+^ T-cell activation. Specifically, there is low- and high-level OX40 expression in resting and activated CD4^+^ T cells, respectively, through T-cell receptor stimulating signaling or the T-cell costimulatory molecule CD28 (Cui et al., 2019; Fromm et al., 2018; Wu et al., 2019). In addition to being activated by specific antigens, T-cell receptors also receive stimulations from mature antigen-presenting cells that express CD80 or CD86, as well as cytokines, such as TNF-α, to enhance the immune response, which causes a sustained chronic inflammatory state (Chometon et al., 2020). We found that 12.8 g/kg of HLJDT downregulated CD28, CD80, CD86, CD326, MHCⅡ, and OX40 expression in lymph nodes. This indicates that HLJDT can regulate antigen presentation by DCs by regulating the expression of the aforementioned proteins.

AD is characterized by abnormalities in innate and adaptive immunity. After antigen presentation by DCs, T cells are activated to secrete various cytokines and chemokines that play an immunomodulatory role to maintain and expand the overall inflammatory response (Yoshida et al., 2020). We found that HLJDT exerts anti-AD effects by reducing the levels of classical Th2 cytokines, including IL-3, IL-4, IL-5, and IL-13, as well as regulating cytokines secreted by emerging T-lymphocyte subsets, including IL17A. IL17A is secreted by Th17 cells and can induce acute inflammation; moreover, it promotes the production of S100 proteins to cause eosinophil- and neutrophil-mediated inflammation. Furthermore, IL17A expression in the skin and serum of patients with AD is associated with disease severity (Nograles et al., 2009; Nomura et al., 2014). IL-33 and TSLP are not secreted by T-cell subsets; however, they are also crucially involved in the inflammatory response of AD. As a bifunctional factor, IL-33 plays a physiological role as a nuclear protein and an endogenous risk signal to exert inflammatory effects in numerous inflammatory skin diseases (De la Fuente et al., 2015). IL-33 mediates signaling through the IL-33 receptor complex formed by ST2 (IL-1RL1) and IL-1RAcP (Korhonen et al., 2019). ST2 and IL-1RAcP are overexpressed in the surface of keratinocytes in skin lesions of patients with AD; moreover, ST2L is selectively expressed in Th2 cells. IL-33 is a strong inducer of the Th2 immune response, which promotes the production of IL-5 and IL-13 (Jurca et al., 2020; Oh et al., 2013). Moreover, IL-33 produced by epidermal keratinocytes can induce ILC2 production from skin tissues and lymph nodes, which further stimulates IL-5 release and induces the occurrence of AD-like dermatitis (Takatori et al., 2018). A literature review indicated that TSLP is a master cytokine that induces inflammation and promotes the Th2 immune response through DC activation and polarization (An et al., 2018; Mazzucchelli et al., 2008; Silva et al., 2017). Another study confirmed that TSLP is associated with AD pathogenesis and is highly expressed in skin lesions from patients with acute and chronic AD (Sano et al., 2013). Taken together, these findings indicate that HLJDT can regulate the antigen presentation function of DCs, which weakens T-lymphocyte activation to exert anti-inflammatory effects.

Pruritus is a main characteristic of AD. GRP and SP are crucially involved in histamine-dependent itch neurotransmission; furthermore, GRP upregulation has been presented in both chronic dermatitis and pruritus mice, which suggests that GRP-expressing nerves have a vital role in mediating pruritus (Tominaga et al., 2009). Moreover, Tirado-Sánchez showed that GRP is correlated with pruritus severity and is implicated in the pathological mechanism underlying AD (Tirado-Sanchez et al., 2015). SP is a neuropeptide that is widely distributed in nerve fibers and can activate Mrgprs and neurokinin receptors related to sensory neurons in the dorsal root, which causes pruritus and nociception (Heimall and Spergel, 2017). Moreover, SP can sensitize TRPV-1 and bind with NK-1 receptors from hypertrophic cell membranes, which induces mast cell degranulation to release histamine and causes pruritus (Ganchingco et al., 2019; Kremer et al., 2014). Histamine is a crucial itch mediator that can induce histamine-dependent itch responses by binding H1 or H4 histamine receptors and activating TRPV-1 channels (Shim and Oh, 2008). TRPV1 channels play a role in histamine H4 receptor-mediated pruritus by activating downstream signaling pathways of histamine H4 receptors in dorsal root ganglion neurons (Jian et al., 2016). IL-31, which is a four-helix-bundle cytokine, is another important itch mediator for AD that functions by binding with heterodimer comprised of IL-31 receptor A (IL-31RA) and tumorigenic M. Moreover, IL-31RA is the key neuroimmune link between Th2 cells and sensory nerve; furthermore, high IL-31RA expression could cause pruritus by activating skin nerve endings (Otsuka et al., 2011; Sonkoly et al., 2006). Oetjen reported that neuronal IL-4Rα and JAK1 are accurate and reliable targets for chronic pruritus and that IL-4Rα or JAK1 deletion in sensory neurons can alleviate chronic pruritus. Furthermore, IL-4 can quickly amplify neuronal sensitivity, including histamine-induced scratching behavior to various pruritogens (Oetjen et al., 2017). Moreover, both TSLP and IL-33 can communicate with cutaneous sensory neurons to promote itch (Liu et al., 2016; Wilson et al., 2013). Our study demonstrated that HLJDT can relieve AD itching symptoms by adjusting GRP, SP, TRPV-1, p-JAK1, HRH4, and IL-4αR expressions in dorsal root ganglion and reducing the release of cytokines, including IL-31. Listed drugs for AD (dupilumab, an anti-IL-4Rα monoclonal antibody, and JAK inhibitors) are aimed at single targets; contrastingly, HLJDT might play an anti-pruritus role in a multitarget and multiway manner. Notably, HLJDT lacked an effect on IgE but reduced histamine levels in AD model mice. Contrastingly, Chen (Chen et al., 2020) showed that HLJDT extract (extracted with 80% anhydrous ethanol) did not mitigate the DNCB-induced increase in histamine and IgE levels in BALB/c. This inconsistency could be attributed to differences in modeling methods and drug delivery methods given that HLJDT contains hundreds of ingredients, with only some regulating histamine levels. Further studies are warranted for clarification.

In this experiment, the positive control was not used for AD owing to there is no AD-specific drugs, and mostly, AD patients use hormone drugs. So far, early clinical and basic research had already demonstrated the effectiveness of HLJDT for AD, and the research of Chen et al. (2020) has confirmed the anti-AD effect of HLJDT, which is consistent with dexamethasone, but the mechanism between them is not the same. Moreover, this study mainly focuses on the HLJDT mechanism for AD. Of course, HLJDT is a traditional Chinese medicine compound, containing hundreds of ingredients, some of which are ineffective or even harmful for AD, and the dose of HLJDT in this experiment is relatively high. In the follow-up studies, to achieve the purpose of simplifying a compound and reducing drug dosage, we will study the pharmacodynamic material basis of HLJDT against AD and make a comparison with the effects of pharmacodynamic material basis and HLJDT or the medicine from HLJDT (such as *Scutellaria baicalensis* and *Phellodendron amurense*). For anti-AD mechanism of pharmacodynamic material basis from HLJDT, some blockers or agonists will be used as positive controls for further research, which also helps in the development of yellow detoxification soup and lay the foundation for clinical application.

Conclusions

In conclusion, HLJDT could exert anti-inflammatory and anti-pruritic effects by regulating the antigen presentation function of DCs, weakening T lymphocytes, and downregulating the expression of indices in the dorsal root ganglion. Future studies should investigate the specific components of HLJDT involved in AD treatment. Our findings provide evidence regarding the theoretical and scientific basis for the clinical treatment of AD using HLJDT.

## Data Availability

The original contributions presented in the study are included in the article/Supplementary Material. Further inquiries can be directed to the corresponding author.
