# Supplementary material for: The Anti-Inflammatory and Anti-Pruritus Mechanisms of Huanglian Jiedu Decoction in the Treatment of Atopic Dermatitis
Source: Front Pharmacol. 2021 Dec 2;12:735295. doi: 10.3389/fphar.2021.735295 (PMC8675233; doi:10.3389/fphar.2021.735295)
Supplement: Supplementary file 1 [file Presentation1.PPTX]

## Slide 1
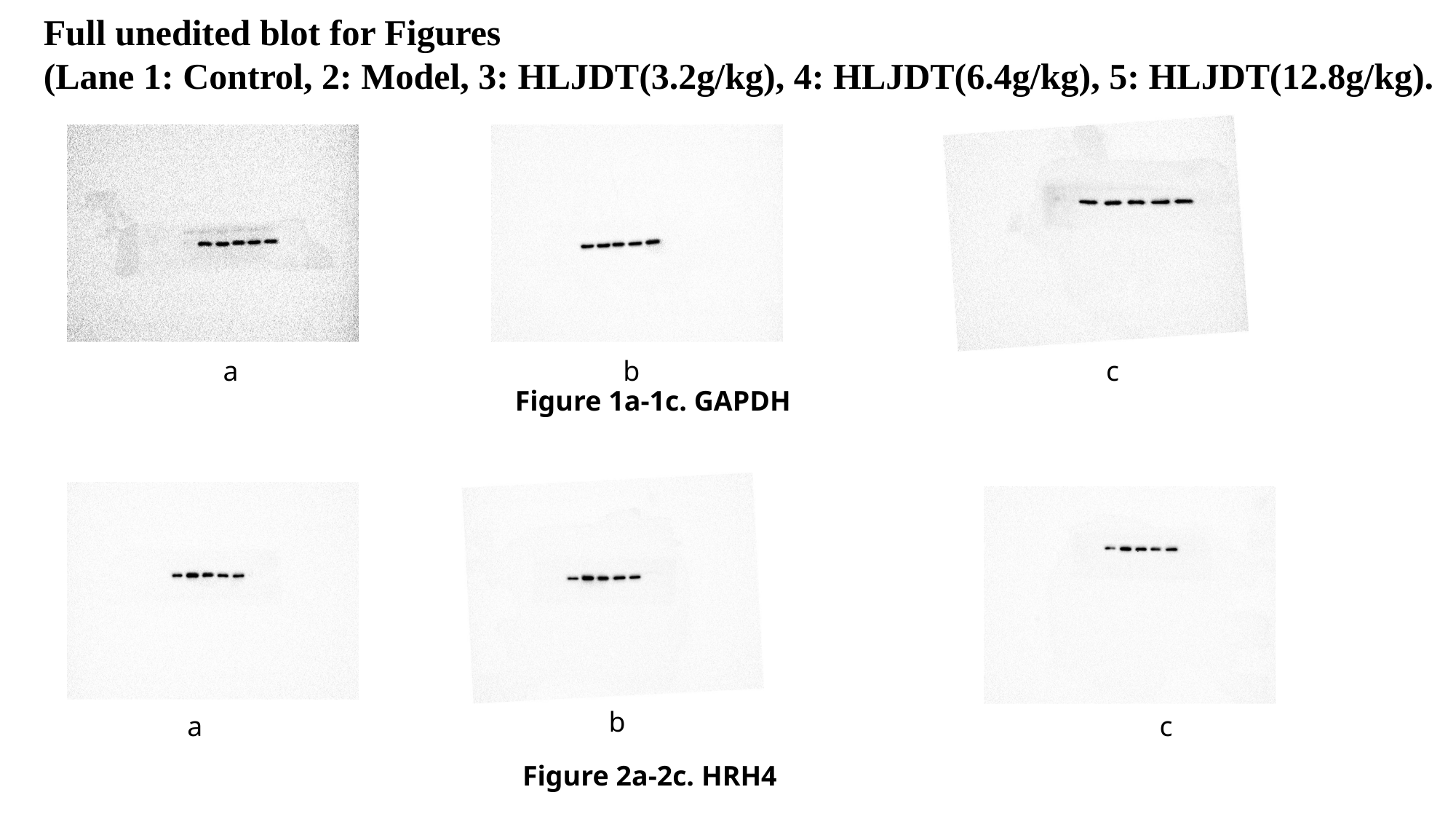

Full unedited blot for Figures
(Lane 1: Control, 2: Model, 3: HLJDT(3.2g/kg), 4: HLJDT(6.4g/kg), 5: HLJDT(12.8g/kg).
a
b
c
Figure 1a-1c. GAPDH
b
a
c
Figure 2a-2c. HRH4

## Slide 2
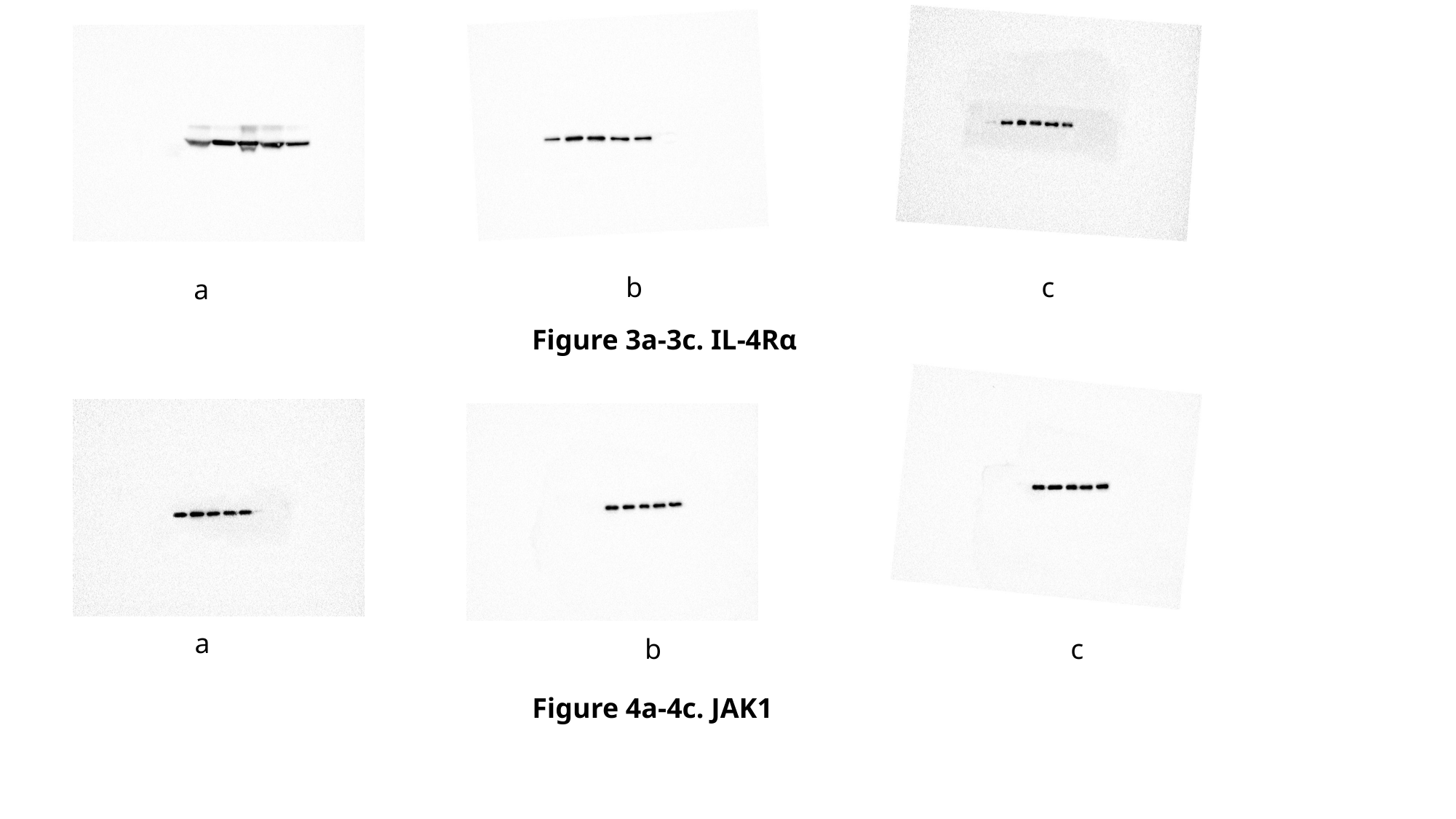

b
c
a
Figure 3a-3c. IL-4Rα
a
b
c
Figure 4a-4c. JAK1

## Slide 3
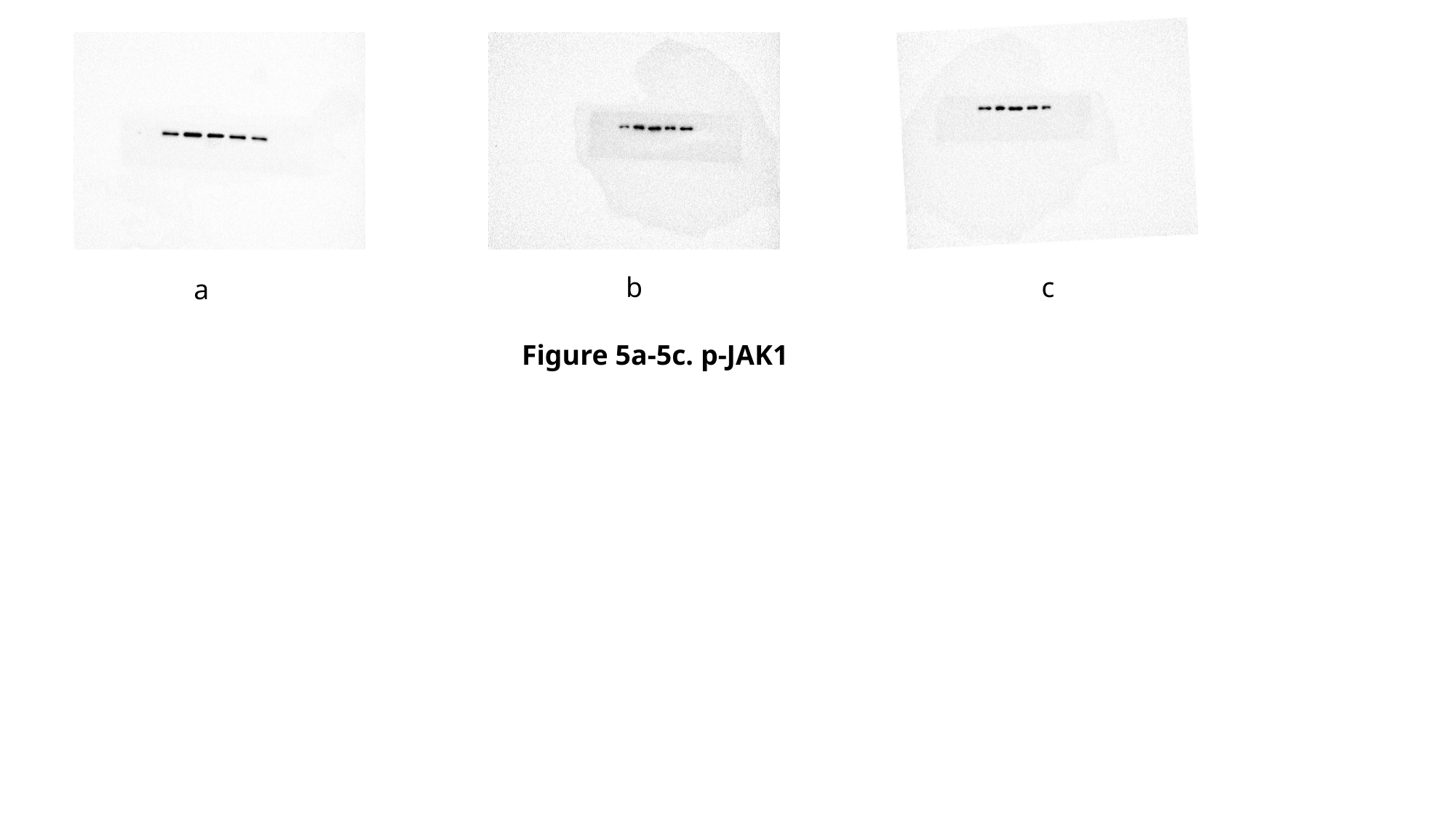

b
c
a
Figure 5a-5c. p-JAK1
