# Supplementary figures and images for: The Anti-Inflammatory and Anti-Pruritus Mechanisms of Huanglian Jiedu Decoction in the Treatment of Atopic Dermatitis
Source: Front Pharmacol. 2021 Dec 2;12:735295. doi: 10.3389/fphar.2021.735295 (PMC8675233; doi:10.3389/fphar.2021.735295)

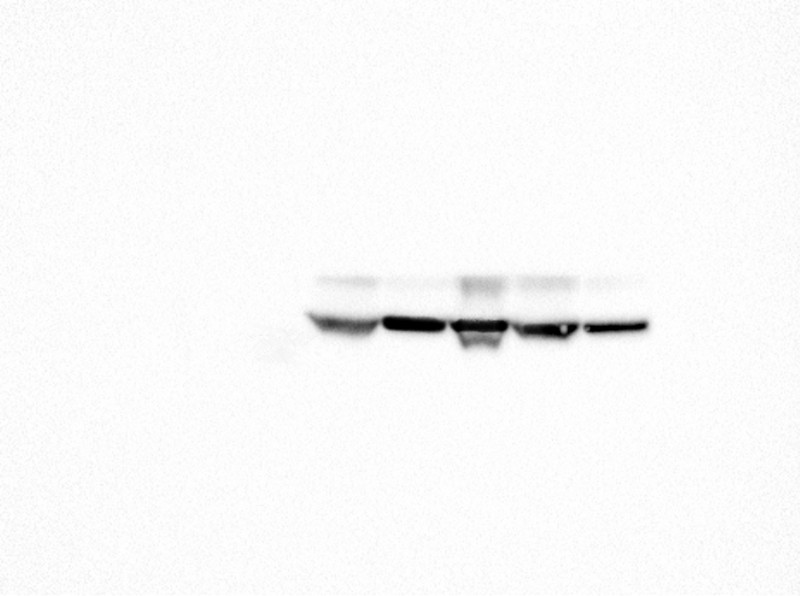

Supplement: Supplementary file 2 [file DataSheet1.zip › IL-4Rα/1.jpg]

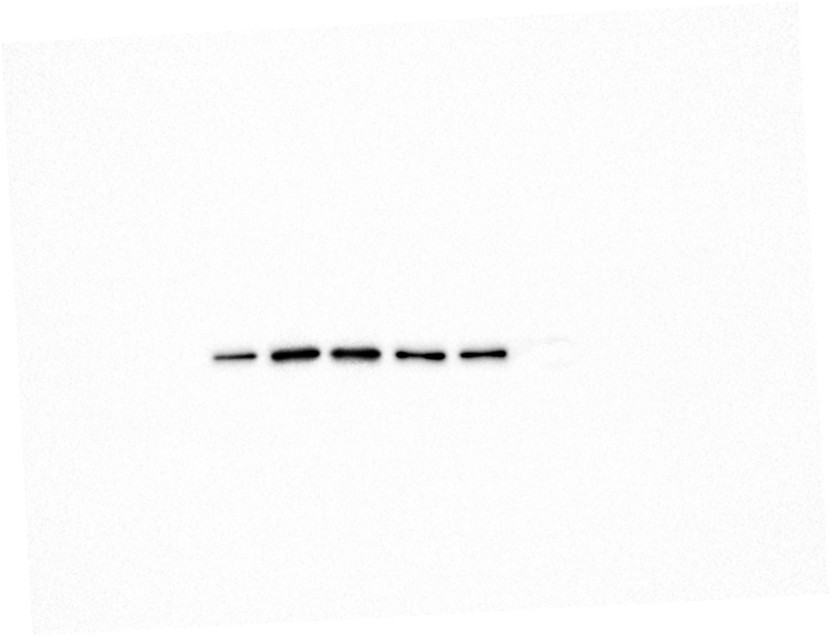

Supplement: Supplementary file 2 [file DataSheet1.zip › IL-4Rα/2.jpg]

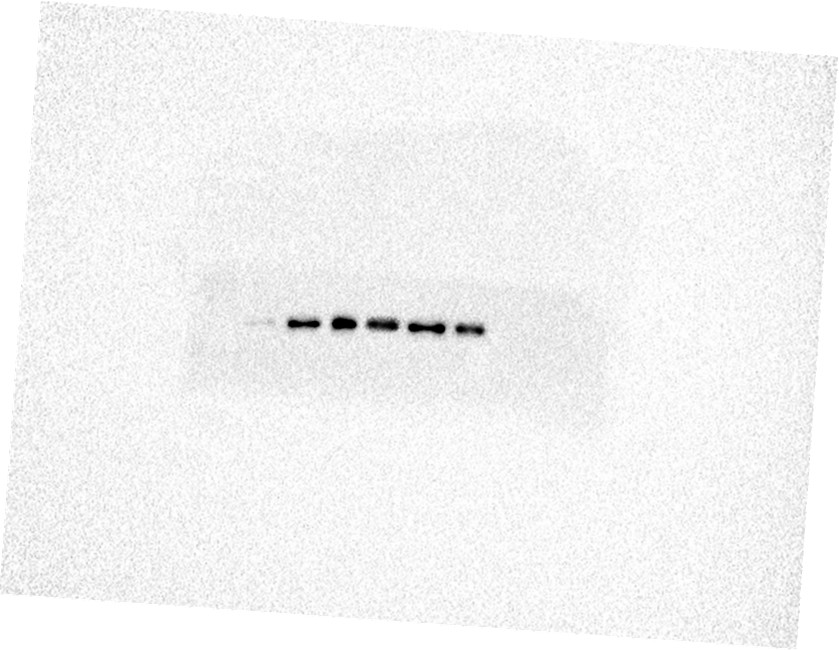

Supplement: Supplementary file 2 [file DataSheet1.zip › IL-4Rα/3.jpg]

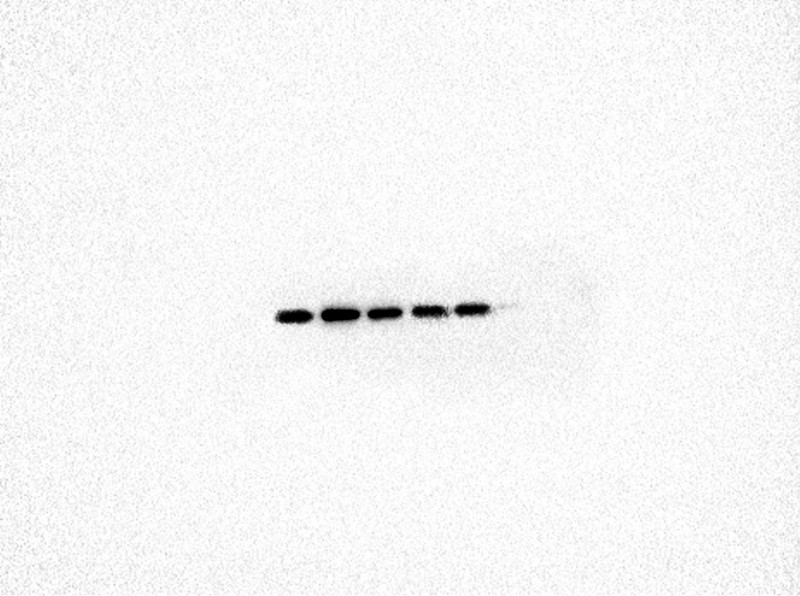

Supplement: Supplementary file 2 [file DataSheet1.zip › JAK1/1.jpg]

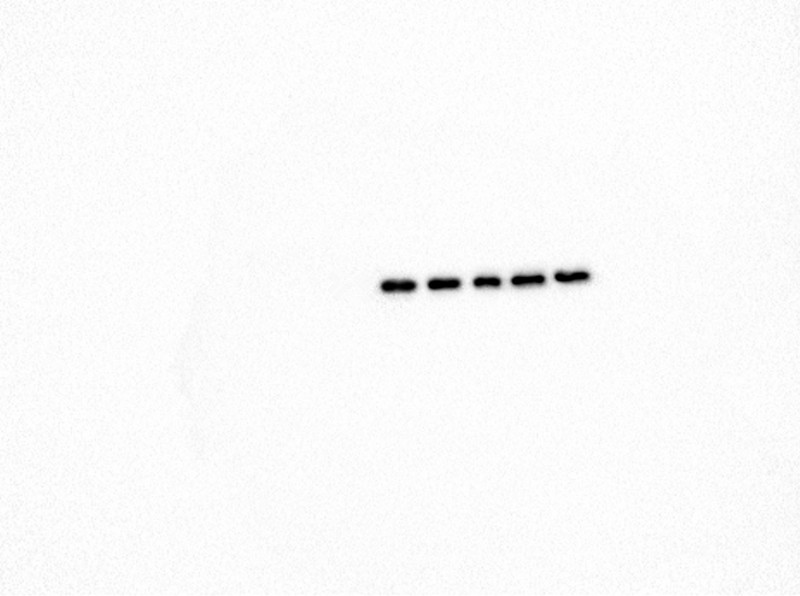

Supplement: Supplementary file 2 [file DataSheet1.zip › JAK1/2.jpg]

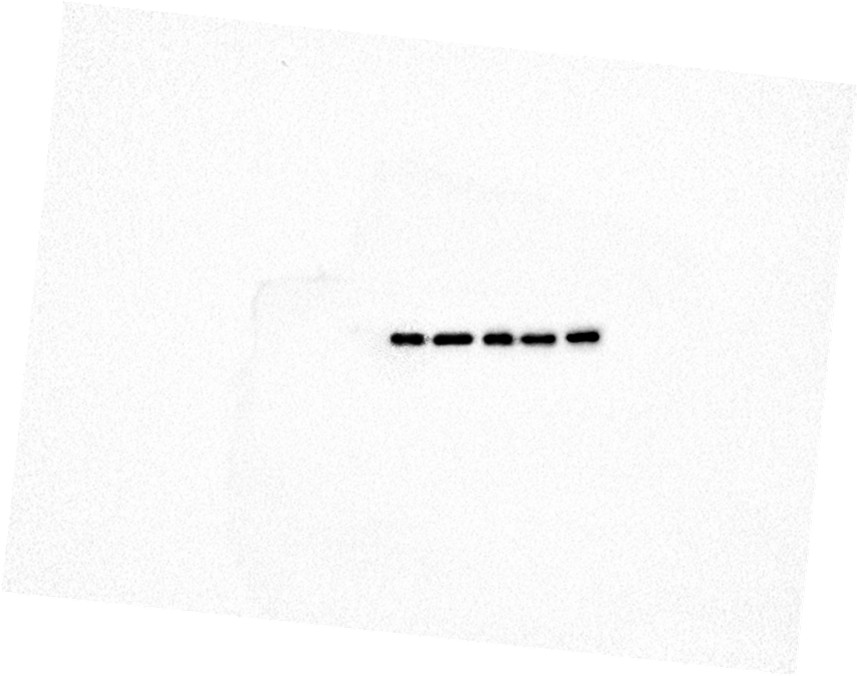

Supplement: Supplementary file 2 [file DataSheet1.zip › JAK1/3.jpg]

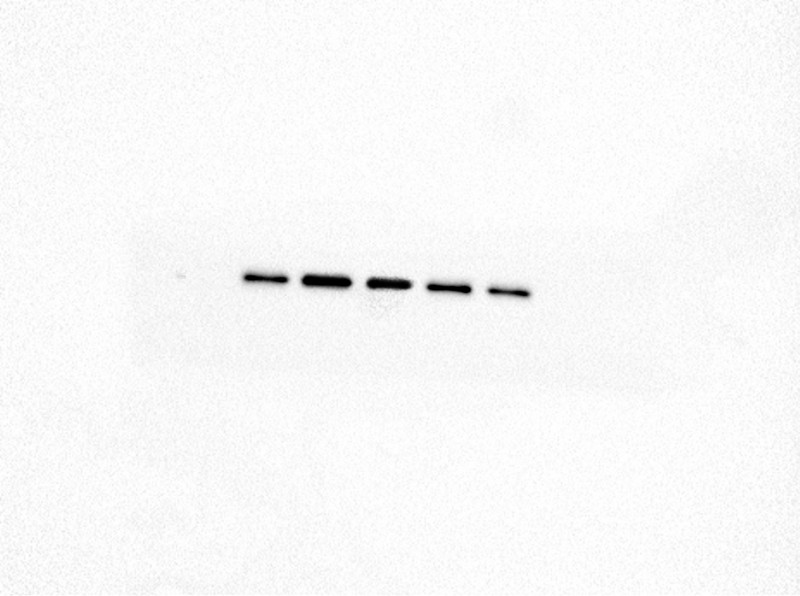

Supplement: Supplementary file 2 [file DataSheet1.zip › P-JAK1/1.jpg]

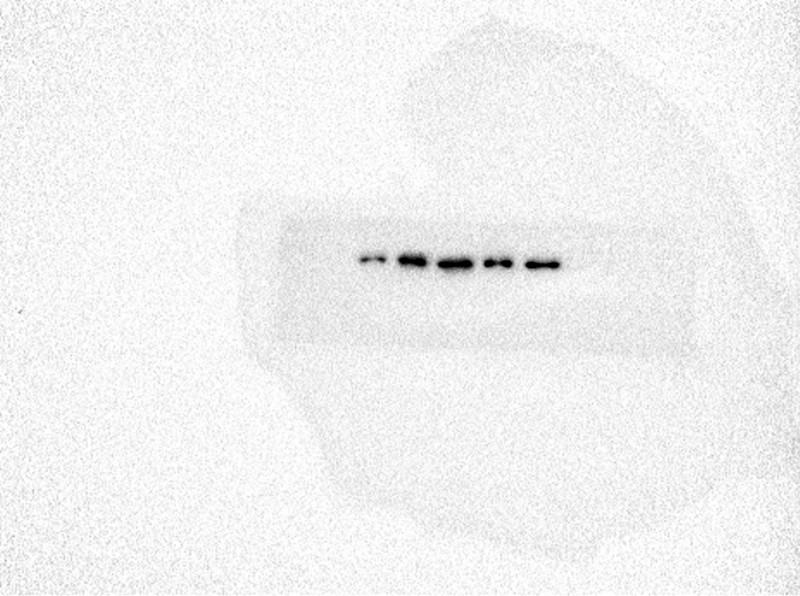

Supplement: Supplementary file 2 [file DataSheet1.zip › P-JAK1/2.jpg]

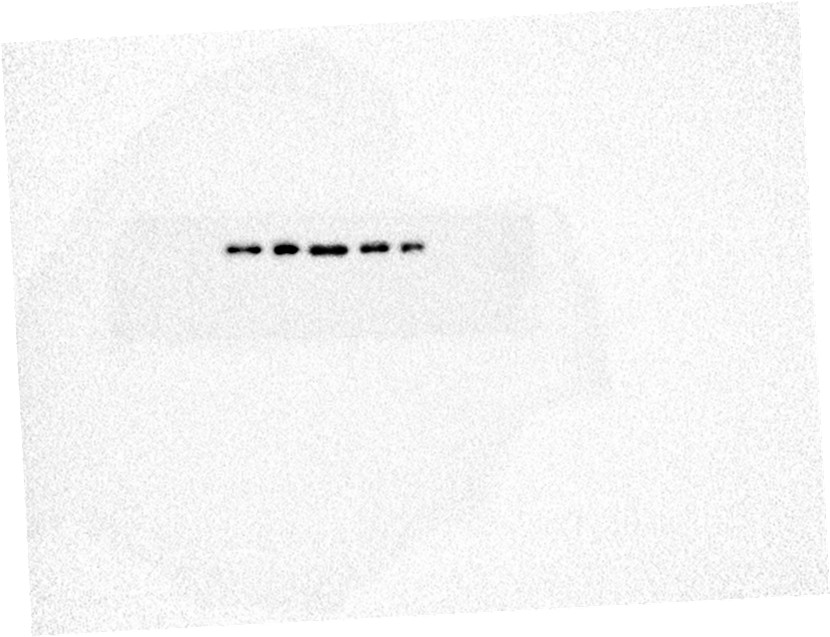

Supplement: Supplementary file 2 [file DataSheet1.zip › P-JAK1/3.jpg]

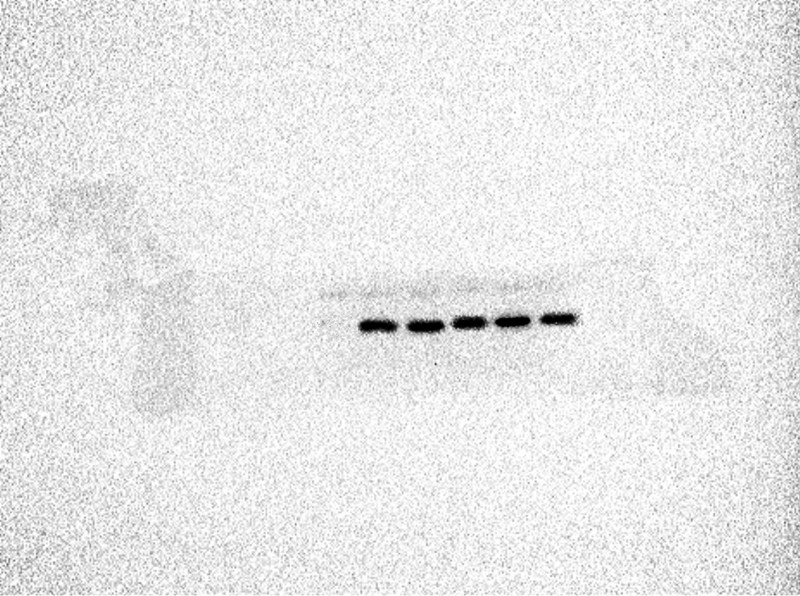

Supplement: Supplementary file 2 [file DataSheet1.zip › GAPDH/1.jpg]

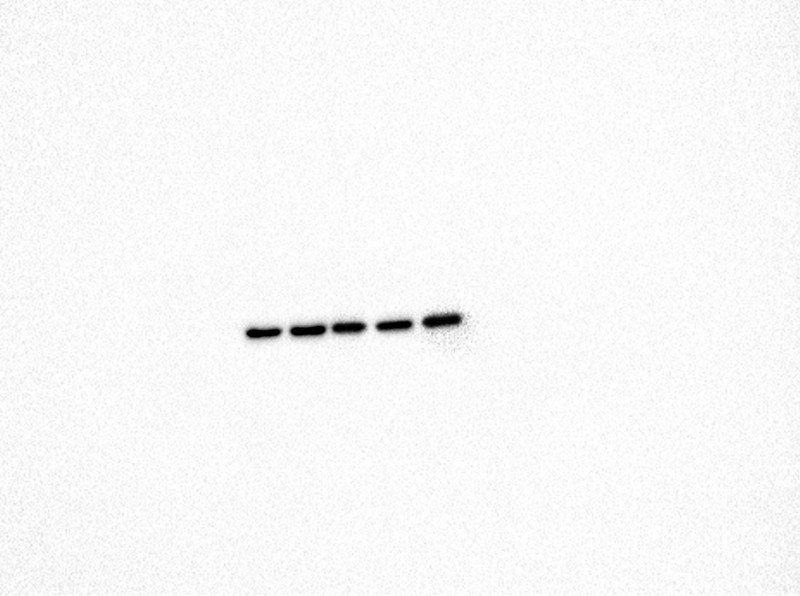

Supplement: Supplementary file 2 [file DataSheet1.zip › GAPDH/2.jpg]

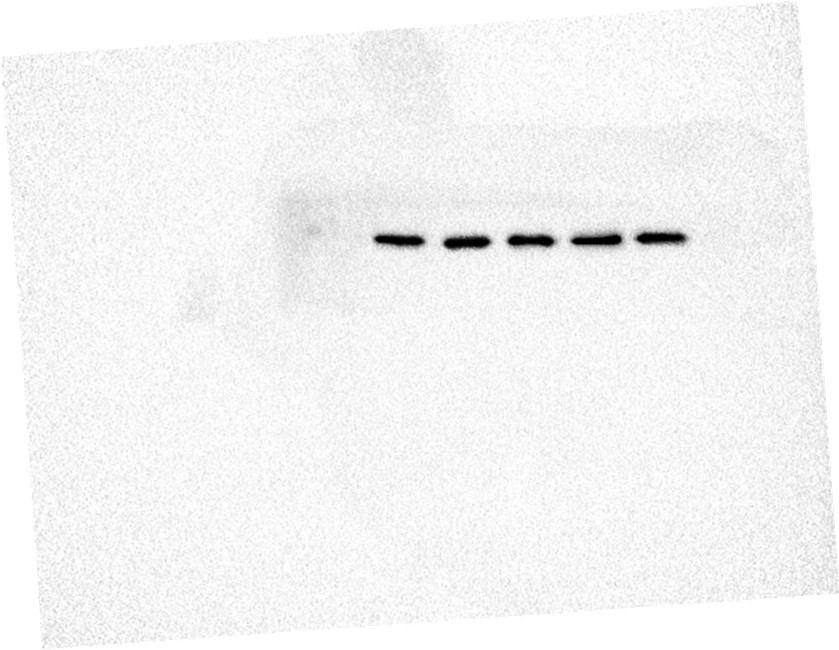

Supplement: Supplementary file 2 [file DataSheet1.zip › GAPDH/3.jpg]

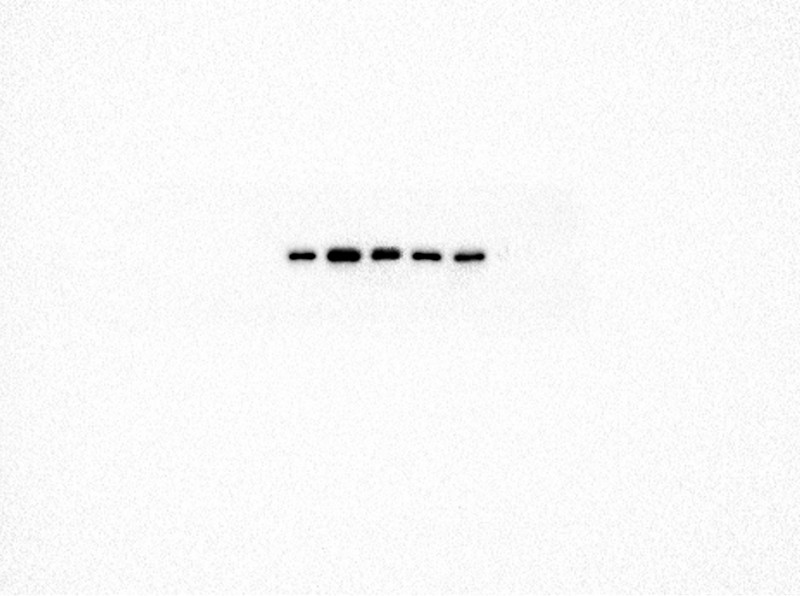

Supplement: Supplementary file 2 [file DataSheet1.zip › HRH4/1.jpg]

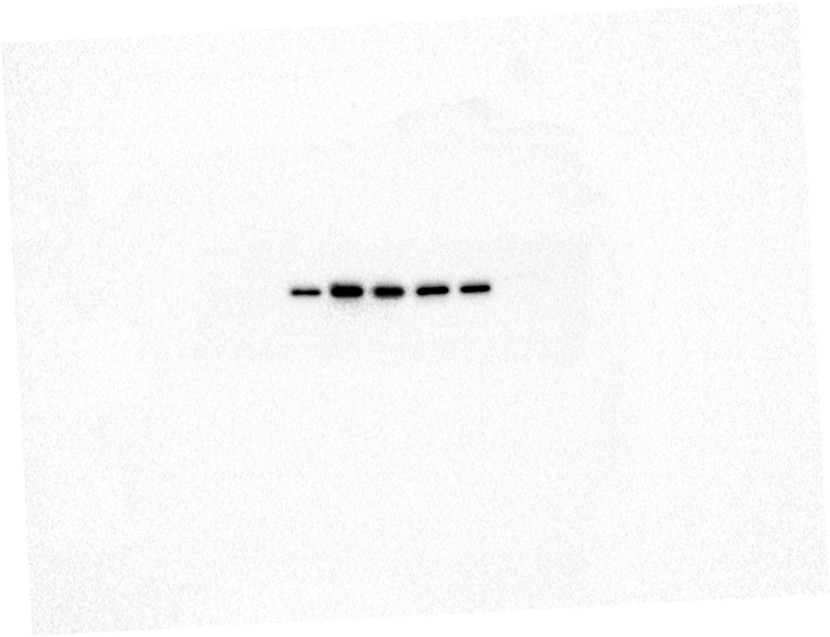

Supplement: Supplementary file 2 [file DataSheet1.zip › HRH4/2.jpg]

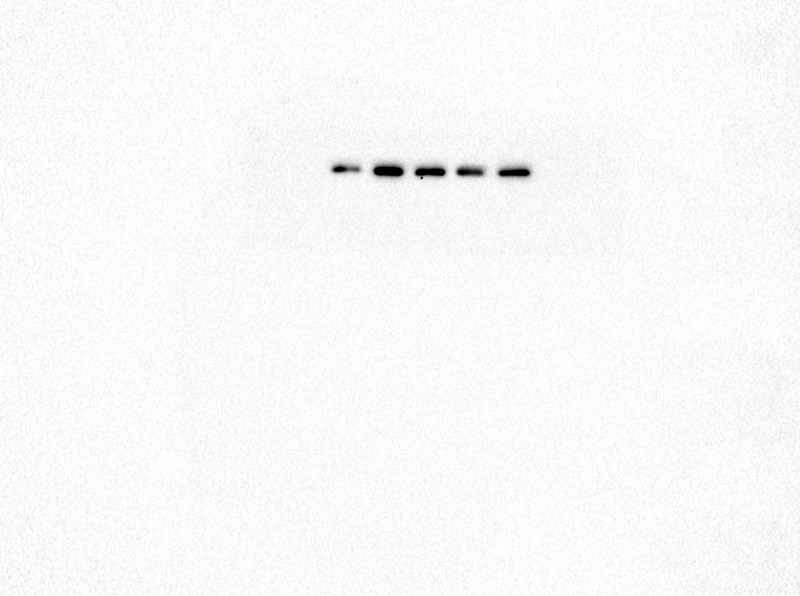

Supplement: Supplementary file 2 [file DataSheet1.zip › HRH4/3.jpg]
